# Transcriptional signature in microglia isolated from an Alzheimer's disease mouse model treated with scanning ultrasound

**DOI:** 10.1002/btm2.10329

**Published:** 2022-05-14

**Authors:** Gerhard Leinenga, Liviu‐Gabriel Bodea, Jan Schröder, Giuzhi Sun, Yichen Zhou, Jae Song, Alexandra Grubman, Jose M. Polo, Jürgen Götz

**Affiliations:** ^1^ Clem Jones Centre for Ageing Dementia Research, Queensland Brain Institute, The University of Queensland Brisbane (St Lucia Campus) Queensland Australia; ^2^ Department of Anatomy & Developmental Biology and the Australian Regenerative Medicine Institute Monash University Melbourne Victoria Australia

**Keywords:** Alzheimer's disease, methoxy‐XO4, microglia, RNA sequencing, transcriptomics, ultrasound

## Abstract

Transcranial scanning ultrasound combined with intravenously injected microbubbles (SUS^+MB^) has been shown to transiently open the blood–brain barrier and reduce the amyloid‐β (Aβ) pathology in the APP23 mouse model of Alzheimer's disease (AD). This has been accomplished through the activation of microglial cells; however, their response to the SUS treatment is incompletely understood. Here, wild‐type (WT) and APP23 mice were subjected to SUS^+MB^, using nonsonicated mice as sham controls. After 48 h, the APP23 mice were injected with methoxy‐XO4 to label Aβ aggregates, followed by microglial isolation into XO4^+^ and XO4^−^ populations using flow cytometry. Both XO4^+^ and XO4^−^ cells were subjected to RNA sequencing and transcriptome profiling. The analysis of the microglial cells revealed a clear segregation depending on genotype (AD model vs. WT mice) and Aβ internalization (XO4^+^ vs. XO4^−^ microglia), but interestingly, no differences were found between SUS^+MB^ and sham in WT mice. Differential gene expression analysis in APP23 mice detected 278 genes that were significantly changed by SUS^+MB^ in the XO4^+^ cells (248 up/30 down) and 242 in XO^−^ cells (225 up/17 down). Pathway analysis highlighted differential expression of genes related to the phagosome pathway and marked upregulation of cell cycle‐related transcripts in XO4^+^ and XO4‐ microglia isolated from SUS^+MB^‐treated APP23 mice. Together, this highlights the complexity of the microglial response to transcranial ultrasound, with potential applications for the treatment of AD.

**Abbreviations**
AD: Alzheimer's disease
Aβ: amyloid‐beta
BBB: blood–brain barrier
FACS: fluorescence activated cell sorting
MBs: microbubbles
NFκB: nuclear factor kappa light chain enhancer of activated B cells
PBS: phosphate buffered saline
PCA: principal components analysis
SUS: scanning ultrasound
WT: wild type

## 1 INTRODUCTION

Alzheimer disease (AD) is the most common cause of dementia worldwide. The disease is characterized by progressive and irreversible neurodegeneration. However, given the complexity of the disease combined with a lack of knowledge on how to treat AD efficiently, there is an acute requirement to develop novel treatment strategies.^1^

At a histopathological level, AD is characterized by the accumulation of extracellular amyloid‐β (Aβ) plaques, intraneuronal tau deposits and increased microglial activation.^2^ A broad range of studies have revealed how microglial cells assume both a protective role (through shielding, recognition, and removal of Aβ) and a detrimental role (through removal of synapses or the release of neurotoxic factors), driving the progression of AD.^3^ Transcriptomic studies on microglia have advanced our understanding of the pathogenesis of AD at the level of transcriptional network dynamics, highlighting important molecular players depending on the different phases of the disease.^4^, ^5^, ^6^ Microglia are known to phagocytose aggregated forms of Aβ, and it has been proposed that deficiencies in this process may contribute to late‐onset AD^7^ and metabolic labeling in humans indicated that clearance of Aβ is impaired in AD.^8^ Recently, it has been shown that Aβ‐containing microglia differ in their transcriptional signature in comparison to microglia that have not internalized the peptide.^9^

An obstacle to treating AD is the blood–brain barrier (BBB), which prevents large molecules such as antibodies from entering the brain, with IgG having 0.1% transfer across the barrier.^10^ Approaches to modify anti‐Aβ antibodies to increase levels in the brain are in development,^11^ along with other approaches to circumvent the BBB.

Studies in animal models of AD have indicated that repeated transient BBB openings that are achieved throughout the entire brain using transcranial ultrasound in a scanning mode together with intravenously injected microbubbles (SUS^+MB^) significantly clear amyloid plaques. One study reported that plaque reduction can occur as fast as 48 h after BBB opening,^12^ and we have shown that this process occurs through microglial phagocytosis.^13^ Ultrasound‐mediated bioeffects (including microglial activation) have also been demonstrated by specifically targeting the hippocampus,^14^, ^15^ but the therapeutic benefit seems to be most pronounced when the brain is treated more globally.^13^ Of note, this clearing process requires BBB opening^16^ and is even effective at reducing Aβ pathology in 22‐month‐old senescent mice.^17^ Combination treatments with ultrasound for delivery of anti‐Aβ antibodies such as Aducanumab that has been recently approved by the Food and Drug Administration (FDA)^18^ or an anti‐pyroglutamylated Aβ antibody,^19^ led to more effective plaque removal and behavioral improvements than in those observed in mice that were treated with either ultrasound alone or antibodies alone.^18^

Ultrasound‐mediated BBB opening has also been achieved in a small safety trial that revealed tolerability in patients with mild AD when a small region of the frontal cortex was targeted.^20^ A subsequent clinical study found that the BBB could be opened in parts of the hippocampus,^21^ with a modest reduction in the amyloid PET signal following three treatments with ultrasound over a 6‐month period.^22^ A recent clinical trial opened the BBB in the frontal lobes bilaterally and resulted in a modest reduction in the amyloid PET signal and significant improvement in neuropsychiatric symptoms.^23^In all these studies, BBB opening by ultrasound was shown to be safe and reversible in that the BBB was fully restored after 24 h.

Several mechanisms have been proposed to explain how BBB opening leads to amyloid plaque reduction, including the uptake of endogenous immunoglobulins^24^ or albumin binding to amyloid,^13^ followed by microglial phagocytosis of Aβ and lysosomal digestion. Here, to gain a better understanding of how the combination of SUS treatment and Aβ internalization affects microglial physiology, we analyzed the transcriptional profile of microglia isolated from APP23 mice (a model of AD) that had been subjected to SUS^+MB^. By using a fluorescent dye to detect Aβ internalization within the microglia, we identified differences between the microglial cells from mice treated with or without ultrasound, as well as between cells that had internalized Aβ or not.

## 2 RESULTS

### 2.1 XO4 and FACS‐based isolation of Aβ‐positive and Aβ‐negative microglia

**FIGURE 1:**
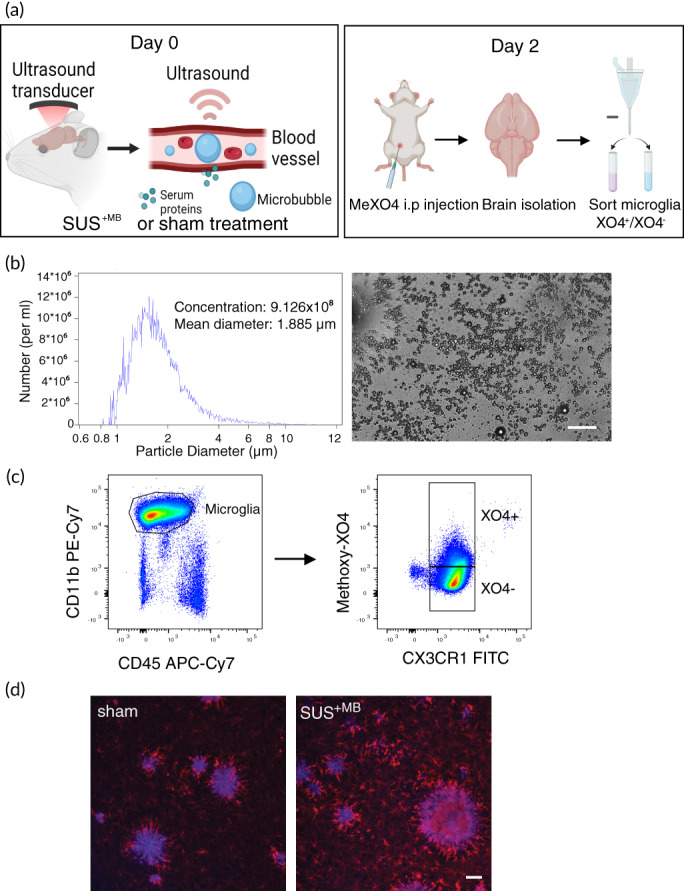
Experimental design and gating strategy to isolate XO4^+^ and XO4^−^ microglia. (a) Scanning ultrasound (SUS^+MB^) or sham (no ultrasound) treatment was applied to APP23 transgenic and wild‐type (WT) mice. Two days post‐treatment, the mice received a single injection with methoxy‐XO4 (that binds Aβ) 2 h before euthanasia and collection of brain tissue. The brains of the mice were harvested and homogenized to form a single‐cell suspension, followed by FACS‐based isolation of XO4^+^ and XO4^−^ microglial cells. (b) In‐house prepared microbubbles were used for scanning ultrasound (SUS^+MB^) and their size and concentration were measured using a Coulter Counter. (c) The gating strategy used to isolate microglial cells into XO4^+^ and XO4^−^ populations via FACS with CD11b and CD45 antibodies to isolate a pure population of microglia, and methoxy‐XO4 fluorescence to isolate microglial cells that contain methoxy‐XO4 bound to Aβ. (d) Methoxy‐XO4 (blue) binds to Aβ plaques in the brains of APP23 mice, with Iba1‐positive microglia in red. Scale bar: 50 μm

To understand the different effects of ultrasound‐mediated BBB opening on plaque‐phagocytic and non‐phagocytic microglia in AD, we applied SUS^+MB^ or sham (i.e., mice were anesthetized and injected with microbubbles but not exposed to ultrasound) to the brains of APP23 mice or WT littermate controls (Figure 1a,b). In addition, to be able to distinguish between microglial cells that had internalized Aβ and those that had not, we used the fluorescent Congo‐red derivative methoxy‐XO4 to stain Aβ within microglia when injected into live mice, as previously done.^9^, ^25^ This allowed us to use a fluorescence activated cell sorting (FACS)‐based technique to separate and isolate XO4^+^ (Aβ phagocytic) and XO4^−^ (non‐phagocytic) microglia following both SUS^+MB^ and sham treatment paradigms (Figure 1c,d).

### 2.2 Genotype, treatment, and Aβ internalization induce distinct phenotypes in microglia

**FIGURE 2:**
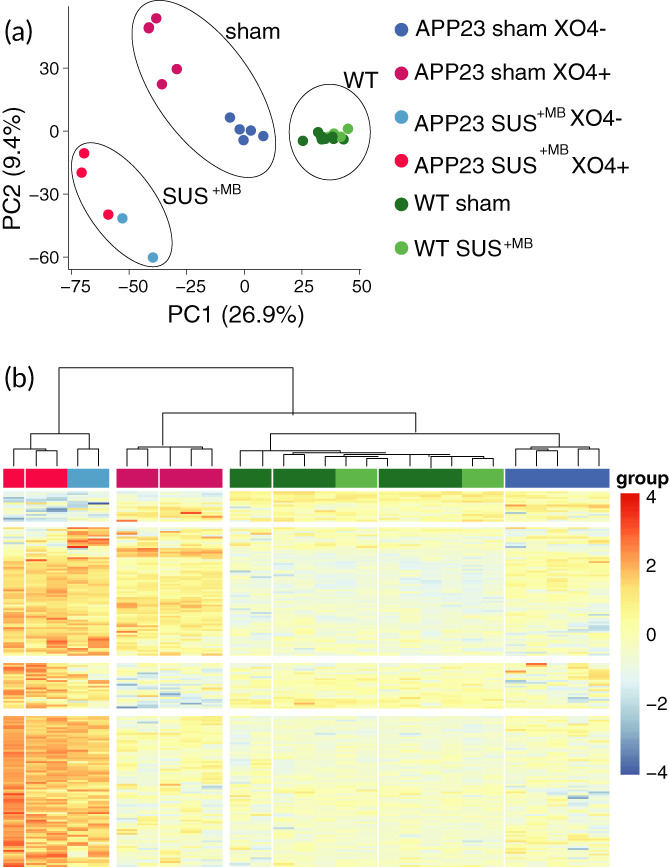
Cluster analysis distinguishes between genotype‐, treatment‐, and Aβ‐dependent microglial phenotypes. (a) Principal component analysis (PCA) reveals the presence of a WT microglia cluster independent of SUS^+MB^ treatment that is segregated from cells of APP23 origin (further clustered dependent on both the SUS^+MB^ treatment and their Aβ content). Ovals indicate treatment groups: SUS^+MB^, sham, and WT mice. (b) Hierarchical clustering of the differential genes (SUS^+MB^ vs. sham) reveals a clear segregation between samples of APP23 or WT origin, SUS^+MB^ treatment or sham, and whether the cells contain Aβ or not (methoxy‐XO4^+^ or methoxy‐XO4^−^).

Sorted microglial cells (both XO4^+^ and XO4^−^) from the four experimental groups (Figure 1a) were subjected to RNA isolation, followed by RNA sequencing (RNAseq) and bioinformatic analysis. Principal component analysis (PCA) revealed a clear segregation between the samples of different genotypes, being either of APP23 mutant or WT origin (Figure 2a). In addition, both PCA and hierarchical clustering analysis (Figure 2b) segregated distinct microglial populations induced by Aβ uptake (XO4^+^ vs. XO4^−^ cells), as well as treatment (SUS^+MB^ vs. sham‐treated animals), which were markedly accentuated in the APP23 samples. Of note, there was no effect of SUS^+MB^ treatment in the microglial transcriptome of WT mice. Thus, we subsequently focused our analysis on the effects of ultrasound ± Aβ internalization in APP23‐derived microglia only.

### 2.3 SUS treatment induces an increased number of up‐regulated genes in microglia

**FIGURE 3:**
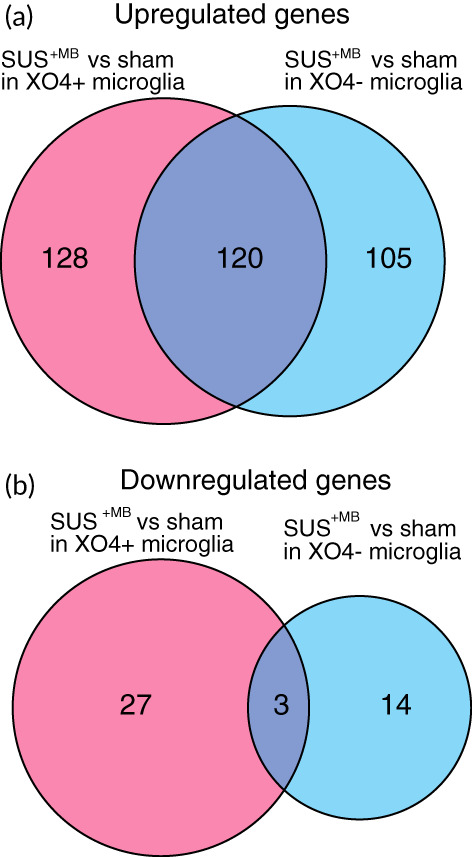
SUS^+MB^ treatment leads to an increase in the number of differentially regulated genes in XO4^+^ microglia, when compared with sham‐treated APP23 mice. (a) A Venn diagram depicting the number of genes up‐regulated by SUS^+MB^ distribute similarly between XO4^+^ and XO4^−^ cells, with many genes up‐regulated in both. (**b**) A larger number of genes were down‐regulated in the XO4^+^ cells compared with XO4^−^ cells following SUS^+MB^, with few genes down in both groups (adjusted *p* < 0.05).

**FIGURE 4:**
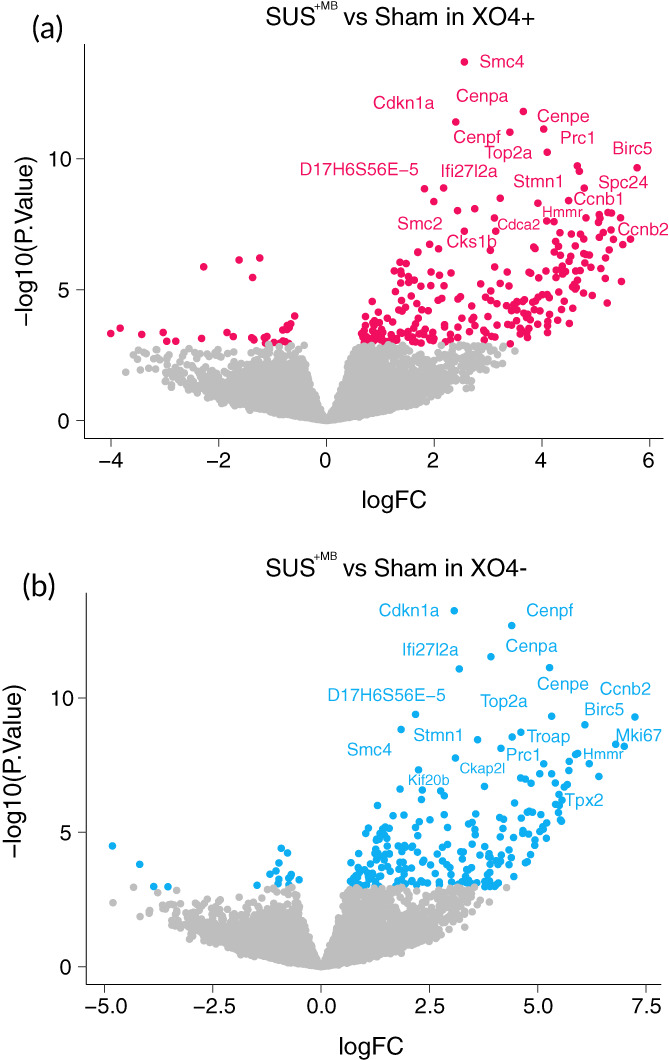
Volcano plots reveal dysregulation of genes in microglia from APP23 mice following SUS^+MB^ or sham treatment. (a) XO4^+^ microglia show a large number of up‐regulated genes. The gene names of the 20 transcripts with the largest fold‐change (logFC) are shown. (b) XO4^−^ microglia also show a large number of up‐regulated genes. The gene names of the 20 transcripts with the largest fold‐change (logFC) are shown.

To gain insight into the response of APP23 microglia to the SUS treatment regime, we further analyzed the transcripts obtained from XO4^+^ and XO4^−^ cells. Our analysis identified 397 differentially enriched genes (FDR ≤ 0.05), with 155 genes being specific for XO4^+^ cells, 199 genes specific for XO4^−^ microglia, and 123 genes being independent of the Aβ signature. Analyzing the treatment‐dependency patterns, we observed that most of the up‐regulated genes were induced by SUS^+MB^, with a total of 353 enriched genes across all the Aβ internalization levels (Figure 3a), and only 44 genes that were down‐regulated following SUS^+MB^ treatment (Figure 3b). These enrichment patterns are highlighted in more detail by the volcano plots, with both the XO4^+^ cells (Figure 4a) and XO4^−^ cells (Figure 4b) exhibiting increased numbers of differentially enriched genes induced by the SUS^+MB^ treatment. The top 50 differentially expressed genes in the SUS^+MB^ versus sham groups for both XO4^+^ and XO4^−^ microglia are presented in Tables 1 and 2 with columns representing log2 fold change (logFC), average log2 expression (AvgExp), moderated t‐statistic, adjusted *p*‐value and B‐statistic for each gene. The B‐statistic is the posterior log odds of differential expression. Log fold change is defined as SUS SUS^+MB^ to sham ratio, with a positive value indicating that the gene is upregulated by SUS^+MB^ treatment.

**TABLE 1:** Top 50 differentially expressed genes in XO4^+^ microglia between SUS^+MB^ versus sham‐treated APP23 mice

Gene symbol	logFC	AvgExp	t	Pval	adj PVal	B
Birc5	5.76502	1.7672889	9.02935	2.22E‐10	3.14E‐07	13.48506
Cdkn3	5.64183	1.5432145	6.744088	1.18E‐07	3.49E‐05	7.515466
Kif11	5.49926	0.2461131	6.576116	1.91E‐07	5.14E‐05	6.97426
Ncapg	5.47342	−0.1786981	5.464827	4.88E‐06	5.93E‐04	3.988828
Cdc20	5.45743	1.0739871	7.403038	1.82E‐08	8.52E‐06	9.133173
Nuf2	5.32476	−0.3289444	6.736156	1.21E‐07	3.49E‐05	7.284114
Ccnb2	5.2893	1.9141487	7.553196	1.19E‐08	6.74E‐06	9.804982
Aurkb	5.28106	0.1698159	7.026936	5.26E‐08	2.05E‐05	8.075848
Cit	5.24623	0.3082589	6.415315	3.04E‐07	7.16E‐05	6.501784
Hmmr	5.22306	1.186993	7.569951	1.14E‐08	6.74E‐06	9.797002
Esco2	5.20983	1.0709507	4.81764	3.25E‐05	2.80E‐03	2.353105
Melk	5.17911	−0.554891	6.172445	6.16E‐07	1.18E‐04	5.824797
Cdc25c	5.17482	−0.7504202	5.556821	3.73E‐06	4.68E‐04	4.188965
Kif23	5.13702	0.6650897	6.945323	6.63E‐08	2.30E‐05	7.956364
Nek2	5.09204	−0.8391415	5.851377	1.57E‐06	2.54E‐04	4.972077
Troap	5.07497	−1.0475974	7.38968	1.89E‐08	8.52E‐06	8.82398
Dlgap5	5.0637	−0.3794831	6.798489	1.01E‐07	3.16E‐05	7.445236
Tk1	5.06171	1.4926731	7.504487	1.37E‐08	7.36E‐06	9.280781
Mcm10	5.04637	−1.0895909	7.260236	2.71E‐08	1.10E‐05	8.355957
Saa3	4.93308	−1.1465763	5.045849	1.67E‐05	1.68E‐03	2.844773
Plac8	4.9074	1.316802	5.889764	1.40E‐06	2.33E‐04	5.218269
Clspn	4.87938	1.3914605	6.258953	4.79E‐07	9.81E‐05	6.258822
Mki67	4.81598	2.6575306	7.401992	1.82E‐08	8.52E‐06	9.451222
Cenpk	4.81418	1.0168275	6.273493	4.59E‐07	9.60E‐05	6.240974
Casc5	4.79524	1.9047316	6.021465	9.56E‐07	1.71E‐04	5.65795
Ccnb1	4.78376	0.8942972	8.35344	1.33E‐09	1.33E‐06	11.79537
Ndc80	4.77516	0.9359373	6.74738	1.17E‐07	3.49E‐05	7.549301
Aspm	4.77431	0.7784471	6.30262	4.22E‐07	8.98E‐05	6.4019
Mns1	4.77059	−0.9456452	5.116901	1.35E‐05	1.40E‐03	2.98948
Stil	4.71375	−0.4286108	6.896241	7.63E‐08	2.46E‐05	7.747276
Ankle1	4.70824	−0.8978755	5.791278	1.87E‐06	2.91E‐04	4.711205
Spc24	4.69279	1.3423116	8.911432	3.03E‐10	3.80E‐07	12.94538
Gpsm2	4.65782	−0.7681863	5.503262	4.36E‐06	5.35E‐04	4.048151
Prc1	4.6556	2.8666675	9.092631	1.89E‐10	3.04E‐07	13.79044
Cenpi	4.63601	0.7782963	5.284361	8.29E‐06	9.00E‐04	3.53967
Rab13	4.6245	−0.436683	5.777597	1.95E‐06	2.91E‐04	4.733482
Cep55	4.61759	0.1648057	6.537217	2.14E‐07	5.62E‐05	6.96793
Hist1h2ae	4.61674	0.6086809	5.236377	9.54E‐06	1.03E‐03	3.448414
Cdc6	4.59923	−0.7363785	5.380778	6.25E‐06	7.20E‐04	3.650384
Foxm1	4.57058	0.2001768	4.66003	5.15E‐05	4.18E‐03	1.887387
Sgol2a	4.56722	0.8256839	5.301333	7.89E‐06	8.65E‐04	3.661214
Tpx2	4.5441	1.3968306	6.899334	7.56E‐08	2.46E‐05	8.057218
Shcbp1	4.5333	−0.2503641	6.219989	5.36E‐07	1.06E‐04	6.048152
Chaf1a	4.5075	−0.3486608	4.197819	1.95E‐04	1.29E‐02	0.699981
Sgol1	4.50239	1.1036653	6.068962	8.32E‐07	1.54E‐04	5.751895
Ckap2l	4.49338	2.7278769	7.949218	3.99E‐09	3.22E‐06	10.8567
Rad51ap1	4.43737	1.7528958	5.761704	2.04E‐06	3.00E‐04	4.897387
C330027C09Rik	4.41274	1.2903056	4.872646	2.77E‐05	2.50E‐03	2.500517
Ccnf	4.38312	0.9642677	5.68638	2.55E‐06	3.43E‐04	4.605822
Ckap2	4.37671	0.4264924	5.925534	1.26E‐06	2.20E‐04	5.33635

*Note*: The logFC changes are reflected by a colour gradient ranging from red (high), to yellow (intermediate) expression.

**TABLE 2:** Top 50 differentially expressed genes in XO4^−^ microglia between SUS^+MB^ versus sham‐treated APP23 mice

Gene symbol	logFC	AvgExp	t	Pval	adj PVal	B
Ccnb2	7.2434	1.914149	8.71212	5.11E‐10	7.22E‐07	12.5665
Mki67	6.99802	2.657531	7.78265	6.32E‐09	4.76E‐06	10.315
Cep55	6.79948	0.164806	7.8447	5.32E‐09	4.30E‐06	9.96164
Nek2	6.41184	−0.839141	6.86382	8.37E‐08	3.50E‐05	7.42704
Hmmr	6.18986	1.186993	7.24982	2.80E‐08	1.46E‐05	8.78338
Birc5	6.08931	1.767289	8.46022	1.00E‐09	1.26E‐06	11.9784
Troap	5.92187	−1.047597	7.56325	1.16E‐08	7.71E‐06	8.95771
Tpx2	5.87434	1.396831	7.53024	1.27E‐08	7.99E‐06	9.61853
Dlgap5	5.72789	−0.379483	7.32031	2.29E‐08	1.29E‐05	8.68108
Stil	5.71934	−0.428611	7.04233	5.03E‐08	2.37E‐05	7.82959
Aurkb	5.68337	0.169816	6.6225	1.67E‐07	5.90E‐05	6.88724
Pbk	5.614	0.578285	6.5491	2.07E‐07	6.86E‐05	6.79667
Ttk	5.56047	−0.003396	6.1612	6.36E‐07	1.75E‐04	5.66954
Fam64a	5.54815	0.172748	5.54325	3.88E‐06	7.30E‐04	4.21982
Bub1b	5.52541	0.852802	5.58235	3.46E‐06	6.62E‐04	4.21705
Aspm	5.49894	0.778447	6.00626	9.99E‐07	2.52E‐04	5.51711
Plk1	5.4907	0.714941	6.32957	3.90E‐07	1.16E‐04	6.2301
Plac8	5.4786	1.316802	5.79718	1.84E‐06	4.24E‐04	4.89829
Kif18b	5.41274	−0.920601	6.03763	9.12E‐07	2.39E‐04	5.27128
Kif23	5.40663	0.66509	6.66974	1.46E‐07	5.45E‐05	7.16429
Top2a	5.32157	3.363108	8.73594	4.80E‐10	7.22E‐07	12.923
Psrc1	5.32146	−1.473526	6.94318	6.67E‐08	2.90E‐05	7.27514
Cenpe	5.27386	3.005189	10.3774	7.47E‐12	1.88E‐08	16.8919
Melk	5.20793	−0.554891	5.49586	4.46E‐06	7.99E‐04	3.98286
Brca1	5.19986	−0.261344	5.04746	1.66E‐05	2.15E‐03	2.80218
Ccnb1	5.13942	0.894297	7.24474	2.84E‐08	1.46E‐05	8.78185
Cit	5.12791	0.308259	5.3118	7.65E‐06	1.17E‐03	3.51658
Ccdc158	5.11777	−1.673486	5.38333	6.20E‐06	1.08E‐03	3.52871
Ndc80	5.07311	0.935937	5.73685	2.20E‐06	4.78E‐04	4.73085
Kif4	5.04925	−0.560068	5.2436	9.34E‐06	1.37E‐03	3.39874
Cenpm	5.04778	0.457076	6.94527	6.63E‐08	2.90E‐05	7.85063
Efcab11	4.93682	−1.413903	4.9978	1.92E‐05	2.38E‐03	2.61281
Sgol2a	4.93232	0.825684	4.83884	3.06E‐05	3.63E‐03	2.39082
Casc5	4.8797	1.904732	5.80704	1.79E‐06	4.21E‐04	5.06427
Cdkn3	4.84763	1.543214	6.66124	1.50E‐07	5.45E‐05	7.39483
Ncapg	4.83529	−0.178698	4.56748	6.73E‐05	6.29E‐03	1.65013
Arfgap3	−4.8143	3.227787	−4.82253	3.21E‐05	3.68E‐03	1.97355
Cdca3	4.8047	1.033934	5.83682	1.64E‐06	4.03E‐04	5.09039
Polr3g	−4.8016	2.483651	−3.08136	4.17E‐03	1.20E‐01	‐1.94254
Nuf2	4.79014	−0.328944	5.16747	1.17E‐05	1.63E‐03	3.13439
Kif11	4.76106	0.246113	5.80768	1.79E‐06	4.21E‐04	4.99501
Pilrb1	4.75962	−0.129751	4.35596	1.24E‐04	1.03E‐02	1.05207
Fxyd6	4.71611	−1.097719	4.32485	1.36E‐04	1.09E‐02	0.93726
Rrm2	4.71417	1.554915	6.78453	1.05E‐07	4.09E‐05	7.67497
Kif14	4.65924	−1.058188	5.27195	8.60E‐06	1.28E‐03	3.28961
Spc25	4.65365	1.777619	5.18905	1.10E‐05	1.56E‐03	3.34028
Omd	4.62121	1.038544	5.63473	2.96E‐06	5.98E‐04	4.39438
Cdca2	4.61168	1.731777	8.22232	1.90E‐09	1.95E‐06	11.4262
Knstrn	4.60604	1.515975	6.81994	9.48E‐08	3.83E‐05	7.75424
Cdc20	4.46665	1.073987	6.07832	8.10E‐07	2.18E‐04	5.73238

*Note*: The logFC changes are reflected by a colour gradient ranging from red (high), to yellow (intermediate), to green (low) expression.

### 2.4 SUS ^+MB^ treatment induces an enrichment in microglial cell‐cycle and phagosome‐related transcriptome

**FIGURE 5:**
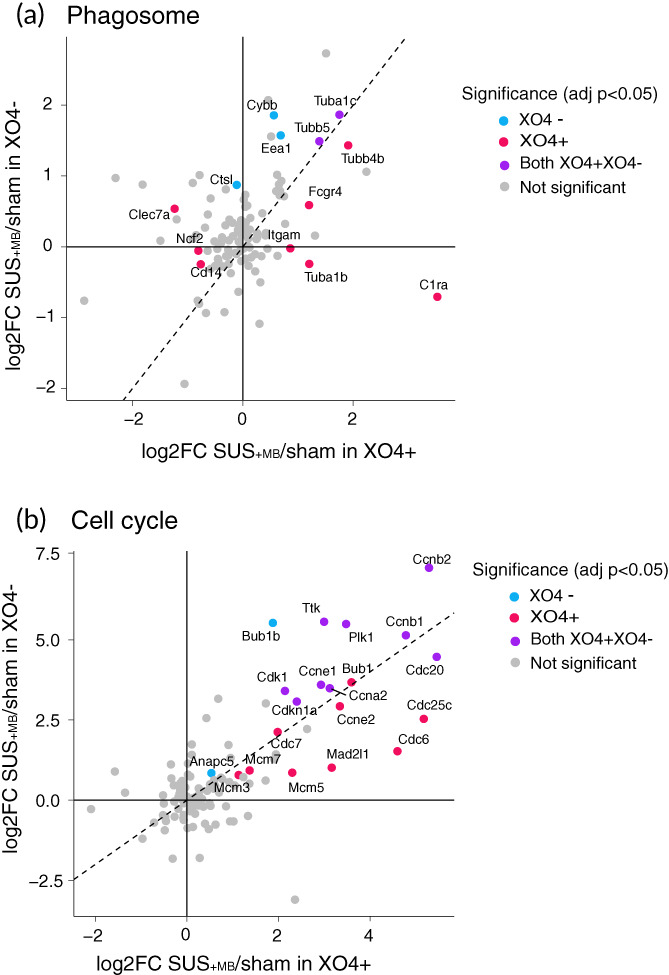
KEGG analysis reveals phagosome and cell‐cycle as top dysregulated pathways in SUS^+MB^ treated APP23 microglia. (a) Plotting fold‐changes in gene expression (log2FC) with XO4^+^ (x axis) and XO4^−^ (y axis) for phagosome genes indicates that the presence of internalized Aβ (XO4^+^) in microglia treated with SUS^+MB^ leads to an additive effect on this pathway. (b) Plotting fold‐changes in gene expression (log2FC) with XO4^+^ (x axis) and XO4^−^ (y axis) for cell cycle genes reveals that they are upregulated regardless of the presence of internalized Aβ (XO4^+^) in microglia, with cell‐cycle genes being enriched in both XO4^+^ and XO4^−^ cells. The significant genes (adjusted *p* < 0.05) are labeled and colored according to their methoxy‐XO4 profile.

We next sought to identify the functionally enriched pathways induced by SUS + MB treatment and compare them in both the XO4^+^ and XO4^−^ microglia. Applying a gene ontology (GO) enrichment analysis to the SUS^+MB^ versus sham datasets revealed the top 10 enriched pathways that included “cell cycle,” “DNA replication,” and “DNA metabolic processes” (Table 3). KEGG pathway analysis revealed that the most enriched pathways included “DNA replication” and “cell cycle,” as well as established pathways in relation to the role of microglia in AD, such as “phagosome” and the “complement and coagulation cascade” (Table 4). Inspection of the “cell cycle” and “phagosome” pathways in a treatment‐ (SUS^+MB^ versus sham) and Aβ internalization (XO4^+^ versus XO4^−^)‐dependent manner revealed similar trends, with a stronger response found for the XO4^+^ microglia containing internalized Aβ (Figure 5a,b). More genes in the phagosome pathway are significantly altered by SUS^+MB^ in XO4^+^ microglia (seven genes up‐regulated and three down‐regulated) than XO4^−^ microglia (five genes up‐regulated) with two of these genes up‐regulated in both (Figure 5a). For the cell cycle pathway, there were also more genes up‐regulated in XO4^+^ microglia (18 genes up‐regulated) than XO4^−^ microglia (11 genes up‐regulated with 9 of these genes up‐regulated in both; Figure 5b).

**TABLE 3:** GO pathway enrichment in microglia from SUS^+MB^‐ versus sham‐treated APP23 mice

	Term	Ont	N	DE	p.DE
GO:0007049	Cell cycle	BP	1204	47	4.25E‐15
GO:0000278	Mitotic cell cycle	BP	657	32	1.12E‐12
GO:0006260	DNA replication	BP	203	19	1.27E‐12
GO:0022402	Cell cycle process	BP	838	36	1.27E‐12
GO:0006259	DNA metabolic process	BP	633	31	2.39E‐12
GO:1903047	Mitotic cell cycle process	BP	544	26	3.37E‐10
GO:0006261	DNA‐dependent DNA replication	BP	113	13	4.33E‐10
GO:0007059	Chromosome segregation	BP	251	18	4.42E‐10
GO:0006974	Cellular response to DNA damage stimulus	BP	618	27	1.07E‐09
GO:0044786	Cell cycle DNA replication	BP	32	8	1.92E‐09
GO:0006302	Double‐strand break repair	BP	187	15	3.05E‐09
GO:0006281	DNA repair	BP	401	21	4.34E‐09
GO:0098813	Nuclear chromosome segregation	BP	196	15	5.82E‐09
GO:0006310	DNA recombination	BP	203	15	9.39E‐09
GO:0000819	Sister chromatid segregation	BP	153	13	1.84E‐08
GO:0033260	Nuclear DNA replication	BP	28	7	2.07E‐08
GO:0000280	Nuclear division	BP	288	17	2.65E‐08
GO:0098687	Chromosomal region	CC	255	16	3.00E‐08
GO:0005694	Chromosome	CC	983	32	3.27E‐08
GO:0048285	Organelle fission	BP	330	18	3.36E‐08

*Note*: The p.DE values are reflected by a colour gradient ranging from white (high) to green (low) p.DE values.

**TABLE 4:** KEGG pathway enrichment in microglia from SUS^+MB^‐ versus sham‐treated APP23 mice

	Pathway	N	DE	p.DE
path:mmu03030	DNA replication	34	6	2.13E‐06
path:mmu04110	Cell cycle	115	9	6.69E‐06
path:mmu00670	One carbon pool by folate	17	4	3.55E‐05
path:mmu04114	Oocyte meiosis	90	5	3.76E‐03
path:mmu05150	Staphylococcus aureus infection	28	3	3.95E‐03
path:mmu04145	Phagosome	110	5	8.73E‐03
path:mmu04914	Progesterone‐mediated oocyte maturation	72	4	9.42E‐03
path:mmu00240	Pyrimidine metabolism	39	3	1.01E‐02
path:mmu03013	Nucleocytoplasmic transport	96	4	2.47E‐02
path:mmu01523	Antifolate resistance	25	2	3.32E‐02
path:mmu03008	Ribosome biogenesis in eukaryotes	68	3	4.34E‐02
path:mmu05322	Systemic lupus erythematosus	29	2	4.36E‐02
path:mmu04610	Complement and coagulation cascades	30	2	4.64E‐02
path:mmu05164	Influenza A	119	4	4.84E‐02
path:mmu03440	Homologous recombination	34	2	5.81E‐02
path:mmu03460	Fanconi anemia pathway	44	2	9.10E‐02
path:mmu05171	Coronavirus disease ‐ COVID‐19	163	4	1.19E‐01
path:mmu01240	Biosynthesis of cofactors	106	3	1.23E‐01
path:mmu05133	Pertussis	53	2	1.24E‐01
path:mmu05140	Leishmaniasis	54	2	1.28E‐01

*Note*: The p.DE values are reflected by a colour gradient ranging from white (high) to green (low) p.DE values.

### 2.5 The magnitude of BBB opening after SUS treatment does not differ significantly between APP23 and WT mice

**FIGURE 6:**
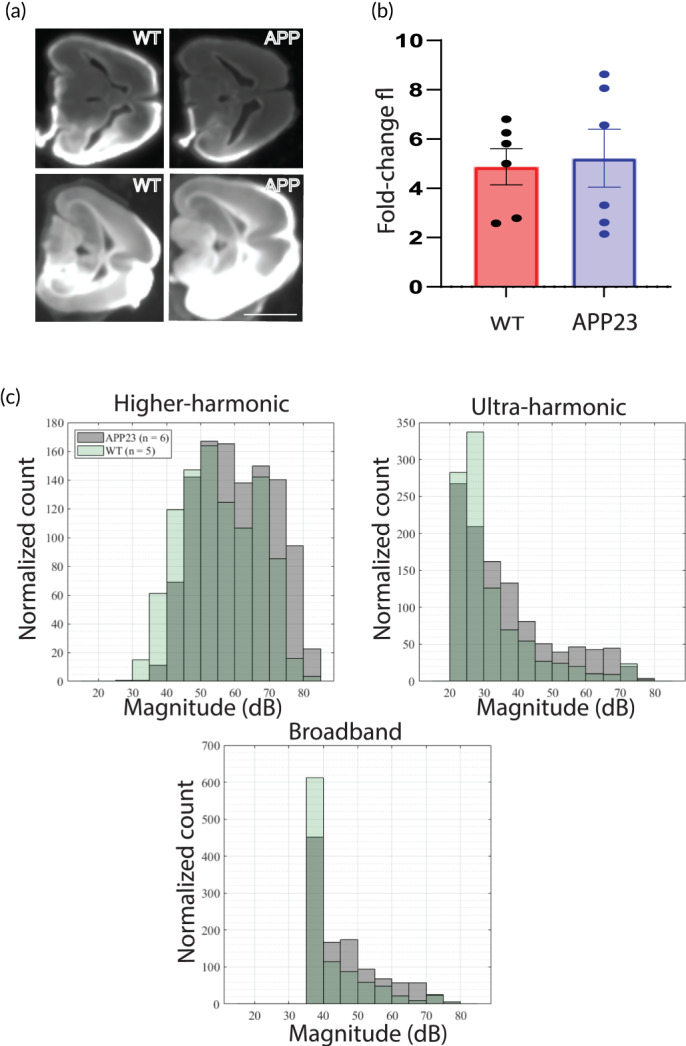
Analysis of BBB opening and acoustic emissions in wild‐type and APP23 mice. (a) Blood–brain barrier (BBB) of WT and APP23 (APP) mice in response to SUS + MB in one hemisphere was visualized by uptake of fluorescently labeled 10 kDa dextran. (b) Quantification of fluorescence in the SUS^+MB^ treated hemisphere compared to the untreated hemisphere reveals a similar fold‐change. (c) Acoustic emissions recorded from WT and APP23 mice recorded reveals an increased higher‐harmonic emission, but similar ultra‐harmonic and broadband emission in APP23 mice compared to WT mice. Scale bar (a): 1 cm

While it was not the major focus of this work, it was surprising to find that there was no effect of SUS^+MB^ treatment on the transcriptome of WT mice at 48 h after treatment while there were many changes in the transcriptome of APP23 mice. To rule out that the differences in the transcriptomic responses between WT and APP23 mice in response to SUS^+MB^ were due to reduced BBB opening in WT mice compared to APP23 mice, we performed an experiment whereby we injected fluorescently labeled 10 kDa dextran as a tracer to quantify the amount of BBB opening 2 h after SUS^+MB^ treatment. The BBB appeared to open similarly in WT and APP23 mice (Figure 6a), and the fold‐change in fluorescent dextran uptake did not differ significantly by genotype (Figure 6b).

### 2.6 The magnitude of cavitation signals recorded during SUS treatment is similar between APP23 and WT mice for ultraharmonic and broadband emissions

We recorded acoustic emissions with a passive cavitation detector (PCD) in order to determine whether there would be less cavitation of microbubbles in WT mice compared to APP23 mice when applying the same ultrasound settings. We extracted different frequency components corresponding to higher harmonics, ultraharmonics and broadband emissions and counted the number of ultrasound pulses of a particular magnitude. Analysis of the cavitation activity recorded during sonication of 20 spots in the right hemisphere from APP23 and WT mice revealed that the former showed an increased magnitude of higher harmonic emissions but that the ultraharmonic and broadband emissions were similar when comparing APP23 and WT mice (Figure 6c).

## 3 DISCUSSION

In this study, we sought to investigate the changes to the microglial transcriptomic profile induced by the application of BBB opening achieved with therapeutic ultrasound in conjunction with intravenously injected microbubbles in a mouse model of AD. This profile was obtained by analyzing the microglial transcriptome and correlating it with the presence or absence of Aβ in the microglia at 48 h after treating amyloid‐depositing APP23 mice with SUS^+MB^. This allowed us to identify several cellular functions that were increased by SUS^+MB^ application in microglial populations that contained Aβ or not, such as the phagosome (reflecting uptake of Aβ for degradation), as well as the cell‐cycle pathway. Therapeutic ultrasound has a relatively larger contribution than Aβ plaque internalization on upregulating transcripts associated with the cell cycle and proliferation and this occurred independently of Aβ internalization. Our findings indicate that BBB opening by ultrasound in the context of AD modulates immunomodulatory functions for at least 48 h.

Previous studies have revealed that BBB opening by ultrasound is a reversible process and that it is fully restored after 24 h as measured by contrast‐enhanced MRI.^23^ Our previous studies have observed a reduction in Aβ plaque load following an ultrasound treatment paradigm consisting of five to seven treatments repeated on a weekly basis,^13^ whereas other studies have observed a response in bulk tissue (including microglia) ranging from 1 week after 6 weekly treatments,^26^ and 6 ^27^, ^28^ and 24 h^27^, ^29^, ^30^ after a single treatment. As microglia are a cell population presenting with a fast and dynamic response depending on both the intensity and time‐point after stimulation (acute vs. chronic response), we sought to observe the transcriptomic changes in these cells outside of the acute response of microglia to BBB opening. We chose a 48 h time point because the BBB is closed by then^13^, ^23^; however, the response of microglia to Aβ plaques was pronounced at this time point as shown by immunohistology,^13^, ^17^, ^24^ and plaque clearance has been shown to peak 2 days after BBB opening as demonstrated by in vivo two‐photon microscopy.^12^

Previous studies have so far investigated the cellular response to ultrasound‐mediated BBB opening in bulk tissue from WT mice and rats and found increased inflammatory transcript levels that were high at 6 h post‐treatment and mostly returned to baseline after 24 h.^27^, ^28^, ^29^, ^31^ Although these studies did not examine changes at 48 h, it would be expected that the inflammatory response would continue to resolve. Along these lines, at 48 h post‐treatment, we have not detected transcriptomic changes in microglia between the experimental groups in WT mice, in stark contrast to the changes observed 48 h post‐treatment in APP23 mice. The long‐lasting effects that we have identified in the APP23 mice could provide an important framework for future AD‐targeted therapies.

Several previous studies have investigated the effect of ultrasound application to the brain by applying *omics* techniques to cell populations. One study investigating ultrasound‐mediated delivery of plasmids to the brain of WT mice performed single‐cell RNA sequencing and found an upregulation of lysosomal genes in microglia 48 h after ultrasound treatment.^31^ In support of this, in a SWATH quantitative proteomics screen following a series of 6 weekly sessions of SUS^+MB^ treatments in aged C57Bl/6 (WT) mice,^32^ we identified an increase in two microglial proteins (LRBA and CAGP) that are involved in phagocytosis.^26^

In our analysis performed a priori to our bioinformatic data mining, we evaluated whether Aβ load (XO4^+^/XO4^−^) and treatment (SUS^+MB^/sham) are independent effects or interacting. Assessing the response of key pathways (phagocytosis and cell cycle), we conclude that the effects are independent and therefore can be analyzed in isolation. Our initial bioinformatics screen aimed to identify differences between microglia from WT and APP23 mice subjected to SUS^+MB^ (with or without Aβ internalization) has revealed several interesting aspects related to the cellular response to the treatment. Thus, the WT microglia revealed the presence of a similar effect on the transcriptome in both sham‐ and SUS^+MB^‐treated experimental groups, as revealed by both the PCA and heatmap analysis. This could be attributed to the fast resolution of microglial response to acute stimulation.^32^ The WT transcriptome was found to cluster in the proximity of the transcriptome specific to XO4^−^ APP23 sham‐treated microglia, reflecting a particular nonphagocytic cellular state, that is, most likely a nondisease associated microglial phenotype. We found microglia from APP23 mice treated with SUS^+MB^ to have more dysregulated transcripts than those from SUS^+MB^ treated WT mice at the 48h time point. One potential explanation is that we were looking at a time point too late to capture transcriptomic changes in WT microglia in response to SUS^+MB^, whereas microglia from APP23 mice remain activated after SUS^+MB^ due to an increased level of microglial responsiveness brought about by amyloid plaques. Another potential explanation for the difference in magnitude of the transcriptomic response between these genotypes could be that SUS^+MB^ induced more BBB opening in the APP23 mice. While we found that there was no systematic difference in the uptake of a fluorescently labeled dextran tracer upon SUS^+MB^ treatment in the two genotypes, there was substantial variability between mice. Future studies should measure the magnitude of BBB opening per mouse by using in vivo MRI imaging at various time points and correlate transcriptomic responses to the amount of BBB opening in each mouse as has been recently done in WT mice.^29^ In addition, the response of microbubbles to ultrasound may differ between APP23 and WT mice because of differences in their cerebrovasculature,^33^ or the fact that APP23 mice weigh less than their WT littermates. We performed recordings of acoustic emissions and found that APP23 mice had higher harmonics emissions than WT mice, but that ultraharmonic and broadband emissions were similar. Broadband emissions are associated with the largest magnitude and most violent cavitation activities, and these were mostly similar between WT and APP23 mice. The cause and significance of this difference in cavitation activity between WT and APP23 mice are unclear; however, it is conceivable that the increased cavitation recorded in APP23 mice might lead to an increased magnitude of transcriptomic changes at 48 h, which warrants further systematic studies, for instance, by using a cavitation controller.^27^

If applied at an early stage of AD, boosting the Aβ phagocytic activity of microglia may present a promising therapeutic strategy by increasing the clearance of protein deposits.^34^ A previous attempt to investigate the microglial response following ultrasound treatment focused on investigating transcripts related to the downstream effects of the NFκB pathway and damage‐associated molecules (DAMs) in bulk lysates from WT rodent brains, with most transcript levels returning to baseline after 24 h.^30^ A subsequent study, however, reported no significant changes in the expression of any of the NFκB‐related genes when using a lower, more clinically relevant dose of microbubbles.^35^ These opposing effects could be attributed to the specific ultrasound parameters that elicit a cavitation‐modulated inflammatory response through the microbubbles present in the blood circulation.^27^ In addition, the transcriptomic response to ultrasound‐induced BBB opening was found to be dependent on the type of anesthesia used during the procedure.^31^ Of note, we used ultrasound settings that we have previously demonstrated to increase microglial phagocytosis,^13^ with no damage to neurons,^36^ and which likely leads to lower levels of cavitation and BBB opening. Taken together, these results indicate that the magnitude of the effects of ultrasound‐mediated BBB opening are dependent on the applied ultrasound parameters, time points and methodology (such as whether RNA or protein levels are investigated, or whether heterogeneous tissue or isolated cell‐types are being analyzed).

Using a protocol that we have recently applied to reveal the transcriptional signature of microglia associated with Aβ phagocytosis,^9^ in the current study, we aimed to investigate changes after SUS^+MB^ treatment in the APP23 model of AD. Our results revealed enriched pathways in the Aβ‐containing microglia, such as cell cycle, phagosome, complement activity, and metabolism. Some of the pathways identified by our analysis have previously been associated with microglial activation in AD, supporting our results. Indeed, microglia have been observed to remove synapses in AD through a mechanism involving members of the complement system.^37^, ^38^ In addition, it has been proposed that the metabolism of microglia is impaired in AD, an effect that can be ameliorated by enhancing the cellular energetic and biosynthetic metabolism.^39^ Increased microglial numbers in the proximity of plaques are associated with more compact plaques and reduced axonal dystrophy,^40^ and we have previously reported increased microglial numbers around plaques following SUS^+MB^ treatment.^17^ Higher numbers of microglia around plaques may result from an increased proliferation or metabolic activity, as hinted at in the present study. Reactivation of the cell‐cycle machinery in microglia following ultrasound treatment is of particular interest, as it has been recently reported that repopulating microglial cells following ablation are neuroprotective in AD.^41^, ^42^

## 4 CONCLUSIONS

We have examined the effect of SUS^+MB^ on the microglial transcriptome in the presence or absence of amyloid pathology. SUS^+MB^ leads to temporary opening of the BBB and alters microglial gene expression in the AD brain to modulate several cellular pathways, including the cell cycle, various metabolic pathways and the phagosome. Harnessing the protective effects of microglia in the context of AD could potentially be achieved by a combination of SUS^+MB^‐mediated BBB opening and targeted drug delivery.

## 5 MATERIALS AND METHODS

### 5.1 Animals

In this study, we have used APP23 mice (harboring the AD Swedish K670M, N671L double mutation in the APP gene^43^) and WT mice. The animals were maintained on a 12 h light/dark cycle and housed in a PC2 facility with ad libitum access to food and water. All experimental procedures in this study (Figure 1a) were approved by the University of Queensland Animal Ethics Committee (AEC) (QBI/412/14/NHMRC and QBI/554/17/NHMRC), and Monash University AEC (17241) and were conducted in compliance with the ARRIVE guidelines (Animal Research: Reporting in Vivo Experiments).

### 5.2 SUS treatment

Microbubbles comprising a phospholipid shell and octafluoropropane gas core were prepared in‐house. 1,2‐Distearoyl‐sn‐glycero‐3‐phosphocholine (DSPC) and 1,2‐distearoyl‐sn‐glycero‐3‐phosphoethanolamine‐*N*‐[amino(polyethylene glycol)‐2000] (DSPE‐PEG2000) (Avanti Polar Lipids) were mixed in a 9:1 molar ratio and dissolved in chloroform (Sigma), after which the chloroform solvent was evaporated under vacuum. The dried phospholipid cake was then dissolved in PBS with 10% glycerol to a concentration of 1 mg lipid/ml and heated to 55°C in a sonicating water bath. The solution was placed in a 1.5 ml glass high‐performance liquid chromatography (HPLC) vial with the air in the vial replaced with octafluoropropane gas (Arcadophta). The microbubbles were activated on the day of the experiment by agitation of the vial in a dental amalgamator at 4000 rpm for 45 s. Activated microbubbles were measured with a Multisizer 4e coulter counter which reported a mean diameter of 1.885 μm and a concentration of 9.12 × 10^8^ microbubbles/ml. These microbubbles were also observed to be polydisperse under a microscope (Figure 1b).

For treatment delivery, an integrated focused ultrasound system (Therapy Imaging Probe System, TIPS, Philips Research) was used. This system consisted of an annular array transducer with a natural focus of 80 mm, a radius of curvature of 80 mm, a spherical shell of 80 mm with a central opening of 31 mm diameter, a 3D positioning system, and a programmable motorized system to move the ultrasound focus in the x and y planes to cover the entire brain area. A coupler mounted to the transducer was filled with degassed water and placed on the head of the mouse with ultrasound gel for coupling, to ensure unobstructed propagation of the ultrasound to the brain.

For SUS^+MB^ applications, mice were anesthetized with ketamine (90 mg/kg) and xylazine (6 mg/kg) and the hair on their head was shaved and depilated. They were then injected retro‐orbitally with 1 μl/g body weight of microbubble solution and placed under the ultrasound transducer with the head immobilized. A heating pad was used to maintain body temperature. Parameters for the ultrasound delivery were 1 MHz center frequency, 0.65 MPa peak negative pressure, 10 Hz pulse repetition frequency, 10% duty cycle, and a 6 s sonication time per spot. The focus of the transducer was 1.5 mm × 12 mm in the transverse and axial planes, respectively. The motorized positioning system moved the focus of the transducer array in a grid with 1.5 mm spacing between individual sites of sonication so that ultrasound was delivered sequentially to the entire brain as described previously.^13^, ^18^ Mice typically received a total of 24 spots of sonication in a 6 × 4 raster grid pattern. For the sham treatment, mice received all injections and were placed under the ultrasound transducer, but no ultrasound was emitted. The time between injecting microbubbles and commencing ultrasound delivery was 60 ± 10 s and the duration of sonication was approximately 3 min (total time from microbubble injection approximately 4 min).

### 5.3 Acute isolation of microglia and FACS

Two hours prior to brain harvest, mice were injected intraperitoneally with methoxy–X04 (2 mg/ml in 1:1 ratio of DMSO to 0.9% [w/v] NaCl, pH 12) at 5 mg/kg. Mice were euthanized by CO_2_ and transcardially perfused with ice‐cold PBS prior to brain extraction. Whole brains, excluding the brain stem, olfactory bulbs and cerebellum, were dissected for microglial isolation. Single cell suspensions were prepared by mechanical dissociation using meshes of decreasing sizes from 250 to 70 μm and suspensions were enriched for microglia by density gradient separation. Briefly, the cell pellet was resuspended in 70% (v/v) isotonic Percoll (1× PBS + 90% [v/v] Percoll), overlayed with 37% (v/v) isotonic Percoll and centrifuged with slow acceleration and no brake at 2000 *g* for 20 min at 4°C. The microglia‐enriched cell population isolated from 37% to 70% interphase was diluted 1:5 in ice‐cold PBS and recovered by cold centrifugation at maximum speed for 1 min in microcentrifuge tubes. The cell pellet was then stained with antibodies to microglial cell surface markers (CD11b‐PE Cy7, 1:200 Biolegend, #101216; CD45‐APC Cy7, 1:200, BD Biosciences # 103116;) for isolation using the FACSAria™ III cell sorter (Figure 1c). CX3CR1 was stained using CX3CR1‐FITC (1:100, Biolegend, #149019). Microglia were defined as live/propidium iodide (PI)^−^ (Sigma‐Aldrich, St. Louis, MO, #P4864), CD11b^+^, CD45^low^, CX3CR1^+^ cells. The XO4^+^ population gate was set using methoxy‐XO4‐injected WT animals. X04^+^ and X04^−^ microglial populations were sorted separately for further analysis.

### 5.4 Immunohistology

APP23 mice were treated with SUS^+MB^ and were perfused with PBS and drop fixed in 4% paraformaldehyde in PBS. They were then cryoprotected in 30% sucrose in PBS and sectioned at 40 μm with a freezing sliding microtome. Microglia were immunostained with anti‐Iba1 antibody (Wako, JP 1:1000) followed by incubation with an anti‐rabbit secondary antibody AlexaFluor 568 conjugate (Invitrogen). Sections were then co‐stained with 10 μM methoxy‐XO4 (Tocris Bioscience) in PBS with 20% ethanol before cover‐slipping. Images were obtained with a spinning disk confocal microscope (Nikon Diskovery) with a 20× objective, acquiring z‐stacks through the entire depth of the section (Figure 1d).

### 5.5 Bulk RNA‐seq and bioinformatics analysis

RNA extraction from FACS‐sorted microglia was performed using the RNeasy Micro Kit (Qiagen, #74004) and RNA quality was assessed using the Bioanalyser (Agilent RNA 6000 Pico kit; #5067–1513). The libraries were prepared using microglia RNA samples with RIN value ≥7.9 as previously described.^9^ Sequencing reads were mapped to the mouse transcriptome reference genome (GRCm38) using STAR (v020201). We then established read counts for each gene using featureCounts (v1.5.2). The bulk RNA‐seq read counts were further analyzed using R (v4.0.2), limma (v3.42.2), and edgeR (v3.28.1). Data handling and plotting were performed using tidyverse (v1.3.0). In detail, we first removed lowly or nonexpressed genes with the *filterByExpr* function, and we calculated TMM (trimmed mean of m‐values) normalization factors to remove composition bias using *calcNormFactors*. To visualize dimensionality reduction of the sequencing data, we first removed unwanted variation in the data with *removeBatchEffect*. The PCA plot (Figure 2a) is using *prcomp* and *ggplot* to visualize the remaining variance in the data. The heatmap (Figure 2b) is plotting the DEGs from either contrast using the wardD2 method and the *pheatmap* function. To determine differentially expressed genes (DEGs), we first determined the gene‐wise variance trends using *voom*. Then, we built a linear model using the *lmFit* function and all combinations of genotype and treatment, batch, and sex as covariates. The Venn diagrams (Figure 3a,b) are generated with the VennDiagram package (v1.7.0). Tables 1 and 2 show the top 50 DEGs for the two contrasts of interest (ranked by absolute log fold change). Pathway and gene ontology enrichments are calculated from the respective DEG lists using the *kegga* and *goana* functions (Tables 3 and 4).

### 5.6 BBB opening with concurrent cavitation monitoring and dextran uptake

For concurrent cavitation monitoring, a ¼" diameter, 3.5 MHz, unfocused single‐element transducer (V3840N‐SU; Olympus NDT Inc., MA, USA) was employed as a passive cavitation detector (PCD) and placed through the central opening of the transmitting transducer. The detected microbubble emission was then amplified (Model 5662; Olympus NDT Inc.) and sampled using a 16‐bit digitizer card (M4i.4421; Spectrum Instrumentation GmbH, Grosshansdorf, Germany) at 31.25 MHz, in synchronization with the ultrasound pulse excitation of the TIPS system.

The recorded PCD signals were processed off‐line using a custom developed MATLAB (MathWorks Inc., MA, USA) script. Three PCD components, higher‐harmonics (HH), ultra‐harmonics (UH) and broadband (BB) emissions were extracted by selecting the frequency bandwidth within the frequency domain as described (with $f_{0}$ = 1 MHz):

- HH: $4f_{0}-30\;\mathrm{kHz}\leq \mathrm{BW}\leq 4f_{0}+30\;k\mathrm{Hz}$ and $5f_{0}-30\;\mathrm{kHz}\leq \mathrm{BW}\leq 5f_{0}+30\;\mathrm{kHz}$
- UH: $3.5f_{0}-30\;\mathrm{kHz}\leq \mathrm{BW}\leq 3.5f_{0}+30\;\mathrm{kHz}$ and $4.5f_{0}-30\;\mathrm{kHz}\leq \mathrm{BW}\leq 4.5f_{0}+30\;\mathrm{kHz}$
- BB: $3f_{0}+30\;\mathrm{kHz}\leq \mathrm{BW}\leq 4f_{0}-30\;\mathrm{kHz}$ and $4f_{0}+30\;\mathrm{kHz}\leq \mathrm{BW}\leq 5f_{0}-30\;\mathrm{kHz}$

A 30 kHz interval was selected by inspecting the frequency spectrum to differentiate the PCD component. Finally, the intensity of selected components was calculated using Parseval's theorem as following:

$$I=\sum_{f\;\text{within}\;\mathrm{BW}}\frac{{\left|S\left(f\right)\right|}^{2}}{N_{\text{FT}}}$$

where $S\left(f\right)$ and $N_{\text{FT}}$ denote the frequency spectrum of the PCD signal obtained by the Fourier transform and the number of frequency bins in the spectrum, respectively. Mice received 15 sonication spots directed at the right hemisphere. Immediately after the sonications, the mice were injected with 20 mg/kg 10 kDa lysine‐fixable dextran‐TMR (Invitrogen) in saline retroorbitally and after 2 h they were perfused with a saline flush followed by 20 ml of 4% paraformaldehyde. Sections were cut with a vibratome at 100 μm thickness and imaged using a LiCor infrared scanner. Two sections per mouse were quantified, comparing the fluorescence signal in the sonicated to the untreated control hemisphere.

## AUTHOR CONTRIBUTIONS

**Gerhard Leinenga:** Conceptualization (equal); formal analysis (equal); investigation (equal); writing – original draft (equal). **Liviu‐Gabriel Bodea:** Conceptualization (equal); formal analysis (equal); writing – original draft (equal). **Jan Schröder:** Formal analysis (equal); writing – original draft (equal). **Giuzhi Sun:** Investigation (equal). **Yichen Zhou:** Formal analysis (equal). **Jae Song:** Data curation (equal); formal analysis (equal); writing – review and editing (equal). **Jürgen Götz:** Conceptualization (equal); formal analysis (equal); funding acquisition (lead); writing – original draft (equal). **Alexandra Grubman:** Conceptualization (equal); formal analysis (equal); funding acquisition; writing – original draft (equal). **Jose M. Polo:** Supervision, Formal analysis (equal); writing – review and editing (equal).

## CONFLICT OF INTEREST

The authors declare that no competing interest exists.

### PEER REVIEW

The peer review history for this article is available at https://publons.com/publon/10.1002/btm2.10329.

## Supporting information


**Appendix S1** DEGs_XO4neg_SUS_vs_sham.

**Appendix S2** DEGs_XO4plus.

**Appendix S3** DEGs_XO4plus_SUS_vs_sham.

## Data Availability

The data that support the findings of this study are available from the corresponding author upon reasonable request. Differential gene expression data are provided as supplementary information.
